# Financial influences and the primacy of patient welfare – an empirical and ethical analysis in German cancer medicine

**DOI:** 10.1186/s12910-026-01450-2

**Published:** 2026-05-06

**Authors:** Julia F. L. Koenig, Birthe Aufenberg, Gereon Brei, Sophia Reitmayer, Sabine Sommerlatte, Wolfgang Greiner, Jan Schildmann, Eva C. Winkler, Katja Mehlis

**Affiliations:** 1 National Center for Tumor Diseases (NCT), NCT Heidelberg, a partnership between DKFZ and Heidelberg University Hospital, Germany, Heidelberg University, Medical Faculty Heidelberg, Department of Medical Oncology, Heidelberg, Germany; 2 Institute for Medical and Data Ethics, Heidelberg University, Faculty of Medicine, Heidelberg University Hospital, Heidelberg, Germany; 3 Department of Health Economics and Health Care Management, School of Public Health, Bielefeld University, Bielefeld, Germany; 4 Institute for History and Ethics of Medicine, Interdisciplinary Center for Health Sciences, Medical Faculty of Martin Luther University Halle-Wittenberg, Halle (Saale), Germany

**Keywords:** Ethics, Health economics, Financial influence, Qualitative research, Cancer medicine, Medical decision-making

## Abstract

**Background:**

Little is known about how financial influences affect medical decision-making and may challenge the primacy principle of patient welfare. First qualitative results in cancer medicine identified specific decision-making situations that can be influenced by financial considerations and characterized by the type of their financial influence. These qualitative findings provide evidence that these influences are largely shaped by reimbursement strategies. Nevertheless, questions on how reimbursement strategies affect medical decision-making, and what their normative dimension is regarding the primacy principle, remain unclear.

**Methods:**

To address the research questions, we conducted an empirical qualitative content analysis according to Kuckartz and an ethical analysis using Ives’ framework of reflexive balancing following the “standards of practice in empirical bioethics research” and the “framework for empirical bioethics research projects”.

**Results:**

The empirical analysis identified six financially incentivized actions: to refrain, to reduce, to deflect, to privilege, to prioritize, and to withhold. These were linked to pre-existing definitions from the normative context: rationing, prioritization, deprioritization, and selection. The ethical analysis showed that a lack of transparency about whether reimbursement strategies implement normative priorities or merely regulate costs makes it difficult to assess their compatibility with the primacy of patient welfare.

**Conclusions:**

The empirical and ethical analyses demonstrate that financial influences, as embedded in reimbursement strategies, can challenge the primacy principle by shaping which options appear feasible or appropriate in practice. Greater transparency and clearer justification of the goals embedded in reimbursement systems are required to determine when such influences are ethically acceptable and how they should be governed.

**Trial registration:**

Not applicable, as the empirical part of this study is based on qualitative interview data and does not constitute as a clinical trial.

**Supplementary Information:**

The online version contains supplementary material available at 10.1186/s12910-026-01450-2.

## Background

When Norman Daniels and James Sabin [1] wrote that “[…] the (financial) incentives also pose novel conflicts of interest with the Primacy Principle […] and thus threaten the trust in doctors”, they voiced a concern that remains highly relevant. The primary principle of healthcare—often summarized as “salus aegroti suprema lex” [patient’s welfare shall be the highest law [2, 3] – raises fundamental questions about how financial influences and considerations might affect medical practice.

While this debate is on-going [4–6], the empirical evidence and ethical analysis of financial influences remain scarce [7–10]. Existing contributions emphasize incentive design [4], warn against physicians becoming ‘double agents’ torn between patient welfare and cost efficiency [9], and call for coordinated system-wide action [6]. Proposals such as organizational ethics approaches have been made, yet practical implementation guidance is limited [10].

Cancer medicine, with its expensive and extensive use of resources, offers good use cases to examine how financial influences affect healthcare [11, 12]. First qualitative results have identified specific decision-making situations in cancer medicine that can be influenced by financial considerations and characterized them by type of their financial influence [13]. These results show that financial influence is largely shaped by reimbursement strategies.

Yet, little is known about how financial influences affect medical decision-making and challenge the primacy principle.

Therefore, this analysis addresses the following research questions:

1. How do different financial influences affect medical decision-making?
2. What is the normative dimension of these affections?
3. Do these affections challenge the primacy principle and how can this challenge be resolved?

## Methods

### Context of the study and prior research

This analysis is part of the ELABORATE project "Mapping and dealing with financial influencing factors in the treatment of cancer patients—A joint project of medical ethics, oncology and health economics". A qualitative interview study was conducted to collect data on which medical decision-making situations are influenced by financial considerations in cancer medicine. From the outset, this qualitative interview study was designed to address two distinct analytical aims based on the same qualitative interview data:

1. A first analysis with a descriptive and categorizing focus, identifying and systematizing situations in cancer medicine in which medical decision-making is influenced by financial considerations. This part has already been published (Please find a detailed description of the interview guide, sampling, and recruitment in additional file 1 and in the respective publication [13]).
2. A second analysis with an explicit ethical-normative focus, which builds on the empirical results of the first part and examines their normative affections regarding the primacy of patient welfare. This second part is presented in this paper.

### Study design

To address the research questions, we conducted a qualitative content analysis and an ethical analysis, following the “standards of practice in empirical bioethics research” [14] and the “framework for empirical bioethics research projects” [15].

### Empirical analysis: qualitative content analysis

**Fig. 1:**
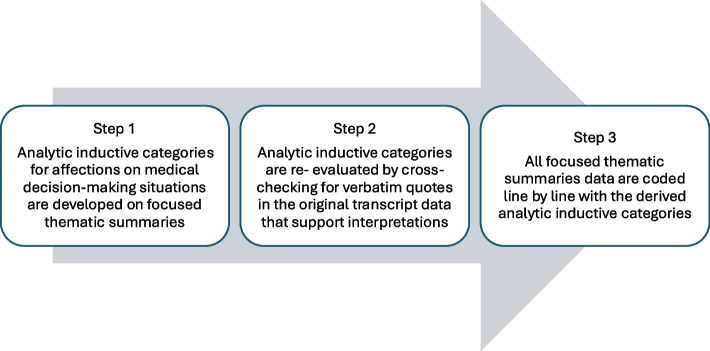
Steps of the qualitative content analysis

To answer the first research question, we conducted inductive qualitative content analysis on the extracted focused thematic summaries based on Kuckartz [16]. All summaries were coded by JFLK with MAXQDA and double-coded by KM for verification and triangulation. Steps of the qualitative analysis are shown in Fig. 1 and coding tree is available in additional file 1. The aim was to extract affections that are representative of the decision-making situations.

#### Rigor

To ensure trustworthiness of the qualitative findings, we applied a range of strategies across data collection, analysis, and reporting, following Adler [17]. Please find a detailed description in additional file 1.

#### Integration of the empirical results and the normative analysis

To address the second research question, we integrated the empirical findings and the normative analysis using the Mapping-Framing-Shaping framework by Huxtable and Ives, which comprises three stages [15]. The first stage (“mapping”) develops a general understanding of the research field; this is provided by the results of König et al. [13]. The present paper encompasses the second (“framing”) and third (“shaping”) stages by focusing on how financial influences on medical decision-making affect patient welfare [15]. To connect empirical results with normative theory, we follow their proposed “bridging methodology” [15].

### Ethical analysis

To address the third research question, we conducted an ethical analysis using Ives’s framework of ‘reflexive balancing’ [18]. This approach is particularly suited to examine tensions between empirically observed action-guiding orientations and established moral principles, such as the primacy of patient welfare.

According to Ives, reflexive balancing seeks to “reconcile tensions” that arise when recalcitrant experiences challenge existing ‘boundary principles’. Boundary principles function as quasi-foundational assumptions—like a null hypothesis—that are treated as provisionally valid for ethical reasoning, without claiming independent epistemic certainty. In contrast, second-order principles are judgements whose justification depends on their coherence with these boundary principles [18].

Patient welfare, as defined by the International Code of Medical Ethics [3], was adopted as the boundary principle due to its broad acceptance in medical ethics. The second-order principle was extracted inductively from the interview data through qualitative analysis of the normative dimension of the reported affections.

**Fig. 2:**
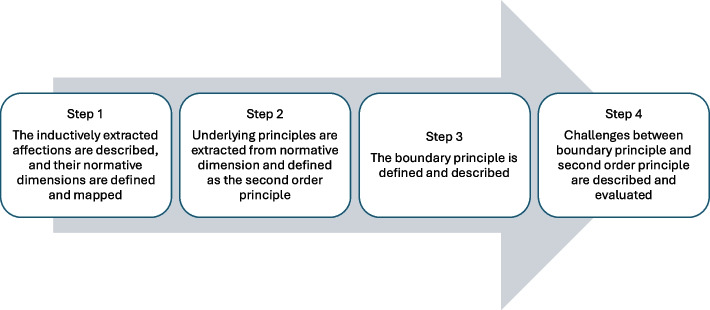
Steps of ethical analysis

Steps of the ethical analysis are shown in Fig. 2.

To avoid conflating descriptive, interpretative, and normative claims, the analysis proceeds on three distinct levels: (1) the descriptive level – what participants report; (2) the interpretative level – how reimbursement strategies are perceived by the participants; and (3) the normative level – the ethical assessment of whether such perceptions are compatible with the primacy of patient welfare.

## Results

### Analytic qualitative results

#### Financially incentivized actions that affect medical decision-making

The interview data indicate that reimbursement strategies, as categorized by the four subcategories of financial influence, profoundly shape the process of medical decision-making, as exemplary quotes show (all exemplary quotes are listed in Table 1). Participants reported modelling patients’ care pathways in line with these reimbursement strategies and perceived this as strongly affecting care, often in ways they did not consider optimal for the patient.

**Table 1:** Quotes from the original data

Subcategories	Participants’ perception	Quote
Financial influence affects medical decision-making	Financial influence affects the process of medical decision-making profoundly	*“[But a single unpaid drug can of course be a really big problem, and there is absolutely no back-up fund that can be used for this.]”* (participant 2) *“[The controller is already calling. He says: “Guys, we're going to have a problem. And then I say: yes, you're right, we're going to have a problem.]”* (participant 5)
	Patient care is affected strongly and participants feel that this is not right	*“[In other words, patients are forced into (…) outpatient care, even though they would have been better treated and monitored on an inpatient basis in terms of their general condition, the disease, complications, side effects of the medication and the need for monitoring, from every medical perspective.]”* (participant 6) *“[(…)some medication that is useful for the patient, but I can't use it because I might not be able to get it reimbursed.]”* (participant 1)
Financially incentivized actions of subcategory (1): ‘no reimbursement’	Incentivized to reduce	*“[(…) That's why we gave our urologist a clear limit on the number of procedures he could perform for years. He was only allowed to perform 250 robotic surgeries because more than that was simply not reimbursed/so (…) or we didn't want to pay for more because it was already quite an expensive affair for us with these disposable instruments and these updates and… and… and. So in way, yes, you can include rationing in this context.]”* (participant 6)
	Incentivized to refrain	*“[Well, I think if you look at it objectively, when it comes to expensive drugs and the introduction of new expensive drugs, there is never any financial incentive. Certainly not for the service provider in the hospital, because at best it is at risk to be reimbursed at all. (…) So I think the incentive is more the other way around, that you don't dare to do it. That's not an incentive, that's fear/That you say, the risk is too high for me, now this, this substance/I don't have a (reimbursement) system for that/The patient actually needs it anyway, and then you try to push it through on an outpatient basis, which might have been better on an inpatient basis.]”* (participant 15)
Financially incentivized actions of subcategory (2): ‘reimbursement, that does not completely cover costs’	Incentivized to reduce and refrain	*“[Sometimes there are also inpatient admissions for social reasons, for diagnostics, for example. That's just something you shouldn't do. But sometimes there's no other option/you have to do a PET CT, MRI, CT/. One reason is that the resources might not be available otherwise. Which is just nonsense in the system. But also because there are really patient-specific reasons why we do it. Because there is simply no other option for the patient. And then they [the cases] are simply cut by the MDK [Medizinischer Dienst der Krankenkassen (The Health Insurance Medical Service)]. That means it's a losing proposition from the outset. Yes, we know that it won't work.]”* (participant 7) *“[So I find bone marrow punctures a very difficult situation at the moment, because we no longer get paid for referrals for bone marrow punctures alone. And our colleagues in private practice also tell us quite clearly: “we get €37 for a bone marrow puncture. For that, I have to stand there for half an hour and then observe the patient. (…) That's not profitable in my outpatient setting. I'd rather do three follow-ups and see other patients and use the time differently and send them directly to you."]”* (participant 9)
	Incentivized to deflect	*“[Smaller hospitals. Smaller departments where it is clearly stated: that is not possible, you cannot do that. Period. And these are mostly patients who need, for example (…) checkpoint inhibitors but are in such a bad condition that they really can't be treated on an outpatient basis and then they say: Sure, but not with us.]”* (participant 5) *“[Financially speaking, if someone presents with fistulae or something like that, they stay for a year, are a piece of work and the bottom line is not much for business. You shouldn’t admit them to your ward in the first place]* “(participant 4)
Financially incentivized actions of subcategory (3): ‘reimbursement, that exceeds the costs’	Incentivized to prioritize	*“[We have understood that oncology is extremely expensive and that it is no longer necessarily just the treatments that are expensive, but also the [diagnostic and organizational] steps towards treatment. This means that we look at every aspect that requires contact [with the patient] here [at the care facility] to see whether certain costs are potentially associated with this case and try to find the most favorable care pathway for the patient and for the hospital, so to speak.]”* (participant 2) *“[There are no infinite resources for medicine, but there is only a finite resource that I need to use as profitable as possible.]”* (participant 1)
	Incentivized to privilege	*“[(…) we don't do that here in our hospital. We usually make a compromise here, which means that we certainly do more than one irradiation fraction, but less than 20 or 25 irradiation fractions. But of course there are colleagues, especially in private practices, who of course have to finance themselves, who exploit this concept of multiple fractions (…) *ad nauseam*, as they say.]”* (participant 3) *“[This is my subjective assessment that this is increasingly leading to people looking at where they can earn the most money. And it's still the case that you earn roughly twice as much from private patients.]”* (participant 17)
Financially incentivized action of subcategory (4): ‘covered costs for treatments with questionable cost-effectiveness’	Incentivized to withhold	*“[There the question really is, is that then significant? Is it two, three years or is it four months, which are statistically significant? If you perform cost analysis on that…] “(participant 5)* *“[Hmm (…), it's really hard to say what's necessary and what's unnecessary. So in my opinion, at least in medical oncology in Germany, chemotherapy is often prescribed for too long.]”* (participant 3) *“[(…) The costs I handle sometimes cause me a bit of stomachache. You notice that when you talk to older people and say, you know, we can't just order the treatment for you on credit. (…) because the therapy costs €6000. Then she [the patient] says: for the whole year? I say: no per bottle (…) and then she says: but my daughter's house isn't being renovated because she's missing €1000, and I'm getting [treatment for] €6000. That's not fair! That also means that people think: yes, what am I actually entitled to?]”* (participant 2)

Across the respective subcategories, the analysis identified distinct patterns of influence, reflected in recurring actions described as consciously or unconsciously incentivized by financial considerations. We therefore termed these patterns ‘financially incentivized actions’, which are summarized below.

Financially incentivized actions of subcategory (1): ‘no reimbursement’:

Participants reported that the performance of non-reimbursed diagnostic and therapeutic measures were reduced or refrained from. For instance, participants reported being disincentivized to use robotic surgery due to a lack of reimbursement. Similarly, they perceived a strong incentive to refrain from prescribing newly approved drugs when reimbursement was still lacking, despite considering them beneficial for patients.

However, participants also described that the above-described measures were performed nonetheless, despite the incentive to refrain or reduce.

Accordingly, subcategory (1) affects medical decision-making by incentivizing the actions to refrain from or to reduce therapeutic or diagnostic measures due to a lack of reimbursement.

Financially incentivized actions of subcategory (2): ‘reimbursement, that does not completely cover costs’:

Similarly to subcategory (1), participants reported that when reimbursement did not completely cover costs of diagnostic or therapeutic measures, they were incentivized to reduce or refrain. Such services were perceived as a financial risk because they were not profitable. Overall, participants suggested that services with insufficient reimbursement, like hospital admissions for diagnostic procedures and administering newly approved drugs to in-patients, were problematic. Quotes from the original data underline that insufficient reimbursement affects decisions on diagnostic procedures and leads to reduce and refrain from diagnostic procedures. Further, participants described an incentive to deflect these measures to other providers, budgets, or sectors, noting that responses depended on providers’ financial situations.

Accordingly, subcategory (2) affects medical decision-making situations by incentivizing the actions to refrain, to reduce, or to deflect diagnostic or therapeutic measures because of insufficient reimbursement.

Financially incentivized actions of subcategory (3): ‘reimbursement, that exceeds the costs’.

In subcategory (3), participants described a strong incentive to privilege or to prioritize certain diagnostic or therapeutic measures (e.g., administering multiple fractions in radiotherapy). They perceived reimbursement as a guideline, signaling which measures should be taken and which should not. When reimbursement exceeded the costs, participants felt that they should privilege or prioritize such measures. Illustrative quotes show how care pathways may be organized around expected cost coverage and how financial considerations are perceived as interwoven with medical decision-making.

Accordingly, subcategory (3) affects medical decision-making by incentivizing the actions to privilege or prioritize therapeutic or diagnostic measures because their reimbursement exceeds the costs.

Financially incentivized action of subcategory (4): ‘covered costs for treatments with questionable cost-effectiveness’.

The focused thematic summary listed in subcategory (4) shows that treatments with questionable cost-effectiveness were perceived as problematic and participant described that they felt an incentive to withhold approved treatment, which costs were completely covered. Participants themselves raised questions of ‘fairness’ and ‘futility’. They perceived it as problematic that high-cost treatments with limited effectiveness were nonetheless reimbursed. The quotes exemplify the insecurity whether it was justified to withhold treatment due to questionable cost-effectiveness regarding overall survival and quality of life. Part of the cost-effectiveness considerations were increasing drug prices in cancer medicine. The narrative structure of the quotes suggests that participants felt that increasing drug prices made questions of fairness more complicated, as illustrated in the quotes.

Accordingly, subcategory (4) affects medical decision-making by incentivizing treatment withholding due to questions of futility and fairness.

In summary, different kinds of financially incentivized actions were derived inductively from the data: to refrain, to reduce, to deflect, to privilege, to prioritize, and to withhold. They all affect medical decision-making and therefore, their normative dimensions need to be analyzed.

### Analytic ethical results

#### Normative dimension of the financially incentivized actions

To understand the normative dimension of the inductively derived empirical results, we examined the extent to which the financially incentivized actions are associated with value judgements, distributional or justice issues. All exemplary quotes are shown in Table 2.

**Table 2:** Normative dimension and definitions of the financially incentivized actions in relation to their respective subcategory of financial influence and the underlining quotes from the original data

Subcategory of financial influence	Financially incentivized actions	Pre-existing definitions from the normative context	Quotes [bold]
(1) no reimbursement	to refrain (from effective and beneficial medical measures)	rationing^+^: withholding effective and beneficial medical measures (e.g. treatment or diagnostic procedures)[19, 20]	*“[(…) So in a way, yes, you can include ****rationing**** in this context.]”* (participant 6)
	to reduce	deprioritization: analogous to prioritization; ranking lower than others	*“[****Sometimes**** there are also inpatient admissions for social reasons, for example for diagnostics. That's just ****something you shouldn't**** do.]”* (participant 7)
(2) reimbursement, that does not completely cover costs	to reduce	deprioritization: analogous to prioritization; ranking lower than others	*“[we get €37 for a bone marrow puncture. For that, I have to stand there for half an hour and then observe the patient. […] ****That's not profitable in my outpatient setting****.]”* (participant 9)
	to refrain	rationing^+^: withholding effective and beneficial medical measures (e.g. treatment or diagnostic procedures) [19, 20]	
	to deflect	selection (lemon dropping): avoiding unprofitable cases [21–23]	*“[You ****shouldn’t admit them**** to your ward in the first place.]“* (participant 4)
(3) reimbursement, that exceeds the costs	to prioritize	prioritization: the practice of structuring priorities in ranking them higher than others [20]	*“[There are no infinite resources for medicine, but there is ****only a finite resource that I need to use as profitable as possible****.]”* (participant 1)
	to privilege	selection (cherry picking): picking profitable cases [21–23]	*“[(…) who ****exploit**** this concept of multiple fractions (…) *ad nauseam*, as they say.]”* (participant 3)
(4) covered costs for treatments with questionable cost-effectiveness	to withhold (from effective and beneficial medical measures)	rationing^+^: withholding effective and beneficial medical measures (e.g. treatment or diagnostic procedures)[19, 20]	*“[Hmm (…), it's really hard to say ****what's necessary and what's unnecessary****.]”* (participant 3)

^+^The classification of “to refrain” and “to withhold” as rationing applies only if the respective diagnostic or therapeutic measure was medically indicated and expected to be effective and beneficial

‘To refrain’ and ‘to withhold’ both describe the evaluative task of rationing. The interview data supports their classification as rationing, provided that the respective measures were medically indicated and beneficial. Quotes underline that a lack of reimbursement is perceived as a macro-level rationing strategy (e.g., robotic surgery), while at the micro level participants describe withholding treatments despite cost coverage (e.g., approved and effective cancer drugs).

‘To reduce’ and ‘to prioritize’ diagnostic and treatment measures are both actions that require ranking their importance. Although reduction is not always equivalent to prioritization, the narrative context suggests this interpretation here. Both terms fall within the normative dimension of priority setting: ‘to reduce’ can be classified as deprioritization and ‘to prioritize’ as prioritization.

‘To deflect’ and ‘to privilege’ are selection strategies whose normative dimension can be described by the colloquial terms ‘cherry picking’ and ‘lemon dropping’, referring to positive and negative selection processes.

In summary, all financially incentivized actions can be related to established definitions from the normative context of allocation and priority setting and are classified accordingly.

Therefore, the financially incentivized actions from the empirical data support the interpretation that participants perceive a normative dimension in reimbursement strategies that can be connected to pre-existing definitions from the normative context of allocation and priority setting. In other words, the subcategories of financial influence acquire their moral character through the perceived financially incentivized actions which can be linked to pre-existing definitions of issues of distributive justice (rationing, selection, prioritization, and deprioritization).

It is important to note that the participants’ perceptions of the normative dimension of reimbursement strategies need to be separated from the participants’ normative evaluations. This manuscript focuses on participants’ perceptions of reimbursement strategies and analyzes the normative implications of these perceptions of patient welfare. The analysis of physicians’ normative evaluation and moral judgment of reimbursement strategies in cancer medicine is beyond the scope of this manuscript.

#### Boundary principle

Patient welfare, the primacy principle, was defined as the boundary principle. Across different formulations in medical ethics, patient welfare consistently expresses the physician’s primary duty to act for the patient’s well-being [24]. In “the principles of biomedical ethics” patient welfare is reflected by the principle of beneficence [25]. For this analysis, we draw on the International Code of Medical Ethics of the World Medical Association, which states that the physician’s primary duty is to promote the health and well-being of patients through competent and compassionate care and to respect their dignity and rights [3]. Patient welfare thus functions as an a-priori value that takes precedence over other interests or duties.

#### Second order principle

The ‘reimbursement strategy’ as identified in the empirical data is not a moral principle in itself but functions as a de facto normative orientation that structures medical decisions. It thus operates as a second order principle in the sense of Ives – an empirically derived guiding principle that may conflict with the moral boundary principle of patient welfare. Verbatim quotes suggest that reimbursement strategies are perceived as a default for organizing patients’ care pathway and as determining medical decision-making (Table 3). But this does not fully explain why a policy instrument should be granted principled status, nor under what conditions this status is ethically defensible. Participants report that reimbursement strategies guide them towards certain diagnostic and treatment measures: measures that are reimbursed appear as those that *should* be carried out, whereas non-reimbursed measures appear as those that *should not* be carried out – regardless of their own clinical judgement (Table 3). In other words: similar to principles, reimbursement strategies are perceived as action-guiding and can therefore be granted principle-status. The analysis of the empirical data underlines that the perceived determination is not limited to decisional dimensions but extends to the normative dimension (compare Table 3). The principle status claim is defensible because the following conditions are met: the empirical findings support the interpretation that reimbursement strategies influence medical decision-making by shaping what is considered feasible or appropriate in a given situation. Thus, reimbursement strategies indirectly influence normative considerations on how clinicians should act. The question of how to act is fundamental to all moral and normative considerations [26]. Principles give guidance on how to act prima facie. Taken together, the empirical analysis and the normative dimension of the financially incentivized actions show that the reimbursement strategies are perceived to give prima facie guidance on how to act. As a result, reimbursement strategies function as an underlying second order principle.

**Table 3:** Quotes that underline the second order principle

Participants’ perception	Quotes
reimbursement strategies are perceived as a default to organize the patients’ care pathway	*“[The general practitioner is satisfied because he gets diagnostics done quickly, which could take weeks or months on an outpatient basis. The patient is satisfied because he also gets diagnostics done quickly. ****From an economic point of view****, I have to say ****that some of the things we do are nonsense****.]”* (participant 9)
reimbursement strategies guide physicians towards certain diagnostic and treatment measures	*[So I think the ****incentive is more**** the other way around, that ****you don't dare to do it****. That's not an incentive, that's fear/That you say, the risk is too high for me, now this, this substance/I don't have a (reimbursement) system for that.]”* (participant 15) *“[This means that ****we look at every aspect**** that requires contact [with the patient] here [at the care facility] to see whether certain costs are potentially associated with this case and try to ****find the most favorable care pathway for the patient and for the hospital****, so to speak.]”* (participant 2)
perceived determination is not limited to decisional dimension but extends to the normative dimension	*“[Sometimes there are also inpatient admissions for social reasons, for diagnostics, for example. ****That's just something you shouldn't do****.]”* (participant 7) *“[There are no infinite resources for medicine, but there is only ****a finite resource that I need to use as profitable as possible****]”* (participant 1)

#### Challenges between boundary principle and second order principle

The boundary principle states that patient welfare should be the a-priori guide for all normative considerations in medicine, including cancer medicine. If normative considerations are a-priori guided by reimbursement strategies and not by patient welfare, then reimbursement as a second order principle challenges the boundary principle. How so: patient welfare as the primacy principle focuses on what is good for the patient in his or her individual situation. Its normative implications are that medical decision-making is guided by what is in line with the patient’s individual welfare [3]. If medical decision-making is guided by reimbursement strategies, it does not necessarily align with patient’s individual welfare. Considerations of cost-effectiveness, proportionality in distribution, and questions of distributive justice might predominate in guiding medical decision-making. While these considerations are normatively relevant at the system level, they shift the focus from the individual patient to the collective of patients.

This shift gives rise to a normative tension: physicians remain professionally committed to the welfare of the individual patient, while reimbursement strategies introduce system-level priorities that may justify limiting or redirecting care. Under these conditions, reimbursement strategies can function as a second-order principle that challenges the primacy principle of patient welfare, particularly when such system-level considerations are not made transparent or explicitly communicated and justified. This raises the question of whether reimbursement strategies may permissibly guide medical decision-making instead of patient welfare.

This question depends on the circumstances and intentions under and for which reimbursement strategies are used. A comprehensive normative evaluation of these factors is beyond the scope of this paper. Instead, we examine which macro-level system conditions must be met for reimbursement strategies to permissibly guide medical decision-making rather than patient welfare.

Questions on how to deal with reimbursement strategies on the micro-level (physician–patient level) are at length addressed in a recent recommendation by the Central Ethics Committee of the German Medical Association (ZEKO) [27]. ZEKO summarizes that physicians should critically reflect on their own actions. It is their responsibility to practice medicine in a way that benefits their patients. Therefore, their a-priori commitment to patient welfare should guide them to responsibly manage financial incentives [27]. For an ethical implementation of reimbursement strategies actors on both the micro and macro level should take and share responsibility.

The empirical results indicate that reimbursement strategies are conceived as guiding normative considerations on how to act: measures judged as important and to be carried out are reimbursed, whereas measures judged as less important are not reimbursed. While this conception does not apply to all reimbursement decisions, it applies in some cases where healthcare systems use reimbursement to pursue health goals, e.g., through disease management programs [28]. Through reimbursement, financial incentives promote these health goals and thereby signal their importance. This importance introduces a normative dimension even though it remains unclear whether these health goals are a priority. Accordingly, incentives implement normative considerations on healthcare goals in the German healthcare system. Thus, at least part of current reimbursement strategies functions as an instrument to implement such normative considerations. Daniels and Sabin make a similar point when they promote accountability of reasonableness for justifying financial incentives in healthcare systems (1): decisions that structure medical decision-making need to be fair and legitimized, like every decision on societal priorities. The underlying considerations behind incentives in a reimbursement system must not remain a ‘black box’. A lack of transparency makes it difficult to understand if reimbursement is used as an incentive or as remuneration. An incentive has a normative dimension; remuneration does not. As long as reimbursement strategies are used as guiding incentives and as remuneration instruments in healthcare systems without accounting for what their function is, no decision can be made whether or not that function is permissible. A normative evaluation of financial incentives is only possible if their function is transparent. Therefore, the challenge that the second order principle ‘reimbursement strategy’ presents to the primacy principle cannot be resolved without transparency.

The lack of transparency needs to be addressed to decide when, and to what extent, reimbursement strategies may permissibly limit patient welfare.

## Discussion and limitations

### Discussion

The results of this empirical and ethical analyses provide answers to the following research questions:

1. How do different financial influences affect medical decision-making?
2. What is the normative dimension of these affections?
3. Do these affections challenge the primacy principle and how can this challenge be resolved?

Six financially incentivized actions which affect medical decision-making in a normatively relevant way were identified: to refrain, to reduce, to deflect, to privilege, to prioritize, and to withhold. The financially incentivized actions were linked to pre-existing definitions from the normative context: rationing, prioritization, deprioritization, and selection. The lack of transparency regarding the implementation of normative considerations through reimbursement strategies presents a challenge to patient welfare.

Discussion of the qualitative analysis of normative implications, the financially incentivized actions and their pre-existing definitions from the normative context.

The results of the qualitative analysis align with empirical data on pre-existing definitions from the normative context of financially incentivized actions in healthcare and cancer medicine.

A comparable German study [7] found that physicians reported being influenced by economic considerations and hospital guidelines in their medical decisions, while chief executive officers denied such effects. Although this study did not examine specific decisions, it supports the notion that financial incentives shape medical decision-making, and its findings can be interpreted using our sub-categories of financial influence and financially incentivized actions.

A growing body of literature exists on most pre-existing definitions from the normative context of financially incentivized actions. While all four pre-existing definitions from the normative context(rationing, deprioritization, prioritization, selection (lemon dropping and cherry picking)) are discussed in the literature, the focus is mainly on rationing [9, 29–32] and prioritization [31, 33, 34]; selection (cherry picking/lemon dropping) and deprioritization are seldom discussed [21–23]. Their extent and effects on cancer patient care remain debated and not fully understood.

Empirical qualitative method cannot show the frequency and extent with which reimbursement strategy challenges patient welfare.

The ZEKO emphasizes that financial incentives are not unethical but require transparency, proportionality, and oversight to prevent over-, under-, or misprovision of care. Hence, the empirical data support ZEKO’s call for greater transparency and for organizational-ethical frameworks that make the underlying normative considerations explicit [27].

#### Discussion of ethical analysis

The argument made for transparency in reimbursement strategy is consistent with other ethical evaluations of financial influences in healthcare. Daniels and Sabin call for accountability for reasonableness in implementing financial incentives (1). Biller-Andorno and Lee conclude that financial incentives must be used carefully because they may undermine trust in the patient–physician relationship (4). They suggest an organizational ethics approach, promoting a “shared-purpose orientation” to align healthcare organizations with “core principals of the medical profession”: patient welfare, patient autonomy, and social justice (4).

Further, Daniels and Sabin argue for a participatory approach that includes as many stakeholders as possible (1). These findings underline the systemic dimension of financial influences on medical decision-making.

Another possible interpretation of reimbursement strategies is that they are deliberately designed to remain normatively ambiguous. From this perspective, reimbursement systems aim to encourage efficiency and cost control at the macro level while avoiding direct involvement in medical decision-making, thereby leaving physicians responsible for specific judgments regarding individual patients. We acknowledge that this interpretation reflects an important and widely accepted rationale for reimbursement systems such as DRGs.

At the same time, our empirical findings suggest that this intended separation of responsibilities is not always perceived as such. Participants described reimbursement structures as shaping what appears feasible or appropriate in specific decision-making situations, sometimes constraining them to act in accordance with their individual patient’s welfare and against their clinical judgment. Such structural ambiguity may have further ethical implications. If reimbursement strategies influence medical decision-making while avoiding explicit normative justification, they may create conditions in which responsibility becomes blurred between macro-level policy design and micro-level clinical judgment. In such situations, physicians may experience moral tension and attribute difficult decisions to the reimbursement framework. Therefore, the results do not negate physicians’ a-priori responsibility to act in accordance with patient welfare at the micro level. Rather, they underscore the ethical relevance of transparency at the macro level: if reimbursement strategies are intentionally designed to foster efficiency while avoiding explicit rationing decisions, then their normative aims, limits, and underlying assumptions need to be communicated transparently. While our data does not allow conclusions about individual motives such as self-deception, the possibility of diffusion of responsibility is ethically relevant. It underlines that ambiguity is not morally neutral: it risks shifting the burden of implicit rationing or prioritization decisions to individual physicians without transparent collective deliberation. This further strengthens the case for transparent normative justification and shared accountability in the design of reimbursement systems.

Further ethical-philosophical analysis is needed to specify what financial influences are permissible and how reimbursement strategies should be appropriately designed.

### Limitations

The analyses have some limitations. A comprehensive ethical analysis of the normative implications of financial influence in healthcare was not conducted because it was beyond the scope of this paper.

All results are limited to medical decision-making in cancer medicine, but the extracted underlying patterns suggest broader relevance. The qualitative analysis is based on interview data that did not reach theoretical saturation of comprehensiveness of all medical decision-making situations that are influenced by financial considerations in cancer medicine according to the definition by Saunders et al. [35]. This was anticipated, as we decided to risk not reaching theoretical saturation in order to gain a stratified and diverse sample and include different perspectives from different federal states, sectors, financial sponsors, and specialties of cancer medicine.

Inductive thematic saturation as defined by Saunders et al. [35] relating to the emergence of new categories, was reached regarding the categories and subcategories of financial influence and financially incentivized actions. The ethical analysis builds on these structural patterns rather than on the completeness of all possible empirical situations. While additional cases might expand the range of examples, they would not necessarily alter the identified normative conflict lines between reimbursement strategies and the primacy principle. It therefore seems unlikely that the limitation of not reaching theoretical saturation presents a bias for the ethical analysis.

## Conclusion

The empirical results of this study indicate that financial influence can challenge the primacy principle. The analysis identifies six financially incentivized actions (to refrain, to reduce, to deflect, to privilege, to prioritize, and to withhold) and four pre-existing definitions from the normative context (rationing, prioritization, deprioritization, and selection (cherry picking/lemon dropping)) and illustrates how they emerge within reimbursement strategies. While these actions are not moral judgments in themselves, the empirical material shows that reimbursement strategies are often perceived as shaping what is practically feasible in medical decision-making. Thereby they define which options appear as “should” or “should not” be carried out in practice. As it is not transparent whether reimbursement strategies are intended to implement normative priorities or merely regulate costs, it remains unclear when and how these systematic incentives limit or support patient welfare. Therefore, the challenge that reimbursement strategy—as second order principle—presents to the primacy principle cannot be resolved.

Further ethical-philosophical analysis and empirical quantitative analysis are needed to address the unanswered questions. 

## Supplementary Information

Supplementary Material 1.

## Data Availability

Due to the risk of identifying participants and the sensitive nature of the discussed topics, original verbatim transcript data remains confidential and will not be shared. The research protocol, analytic coding trees and a German version of the interview guideline are available at request. Data sharing requests can be sent to julia.koenig@med.uni-heidelberg.de. Data access agreements need to be signed to gain access.
