# Supplementary material for: Financial influences and the primacy of patient welfare – an empirical and ethical analysis in German cancer medicine
Source: BMC Med Ethics. 2026 May 6;27:100. doi: 10.1186/s12910-026-01450-2 (PMC13188408; doi:10.1186/s12910-026-01450-2)
Supplement: Supplementary file 1 — Supplementary Material 1. [file 12910_2026_1450_MOESM1_ESM.docx]

**Supplement 1**

Prior socio-empirical research results from the ELABORATE project on decision-making situations in cancer medicine that are influenced by financial considerations:

A qualitative semi-structured interview study was conducted to collect comprehensive, participant-driven data on which medical decision-making situations are influenced by financial considerations in cancer medicine [13]. Interviews took place between February and August 2023 and lasted between 20-90 min [13]. N=45 providers were contacted via e-mail, n=16 interviews were conducted with n=17 participants. [13]. Recruitment, participant characteristics, and sampling strategies are summarized in figure 3.


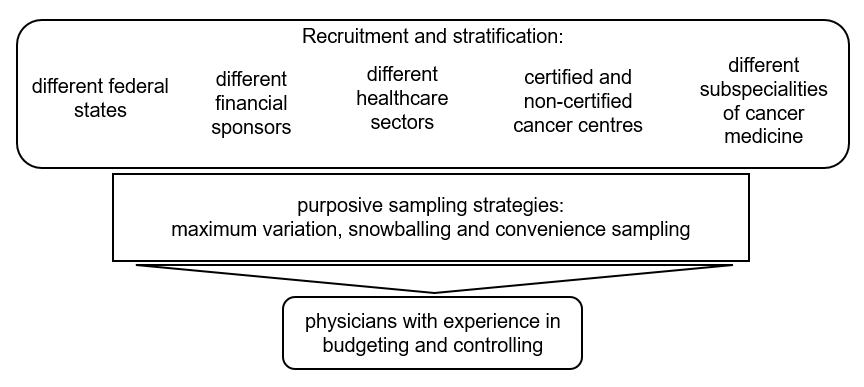


Figure 3**:** Recruitment, participant characteristics, and sampling strategies for semi-structured interview study

Interviews took place at the participants’ workplace with only the participants and the interviewer present [13]. They were semi-structured by an interview guideline, which was adapted after n=5 interviews [13]. The interview guideline consisted of open questions on the influence of financial considerations on medical decision-making in cancer medicine. The interview guideline started with an open and general question on financial considerations and its influence in cancer medicine, followed by more specific questions on the influence of financial considerations on medical decision-making and specific example, including positive and negative aspects [13]. We conducted no repeat interviews and transcripts were only made available to participants on request [13]. Interviews were audio-taped without field notes and transcribed verbatim [13].

To extract medical decision-making situations that were influenced by financial considerations and characterize financial influence a deductive and inductive qualitative content analysis according to Kuckartz was performed [16]. Interviews were transcribed verbatim, deductively coded and medical decision-making situations were extracted as focused thematic summaries. To characterize financial influence, focused thematic summaries were inductively analyzed [13].

N=21 specific decision-making situations in cancer medicine were found and n=4 subcategories of financial influences all related to reimbursement strategies, were characterized: (1) no reimbursement, (2) reimbursement that does not completely cover costs, (3) reimbursement that exceeds costs, and (4) costs covered for treatments with questionable cost-effectiveness [13].

Theory

The ELABORATE project is based on the assumption that financial influences in medicine are justified to a certain extent due to limited resources in the healthcare system and the resulting need for efficiency. [27]. To avoid the misleading and often negatively connotated term “economization” [37] and to include rational considerations on the allocation of resources in medicine, the ELABORATE project instead focused on the influence that financial considerations have or may have on medical decision-making.

Reflexivity

This qualitative empirical-ethical analysis is based on interview data from a study with physicians who have experience in budgeting and controlling. The female interviewer (JFLK) was trained as a physician (internal medicine; hematology and oncology) and worked as a physician part-time. Therefore, detailed and specific explanations of the financial and economic influence on medical decision-making situations in cancer medicine were possible, as there was no need to explain the medical context to the interviewer. Social desirability bias was reduced because the interviewer belonged to the same peer-group as the interview participants but was lower in the professional hierarchy and female. Saturation could not be reached, as we chose to recruit a diverse sample to include many different perspectives from different providers, healthcare settings and sponsors. No prior relationship was established, but there was a prior working relationship with two participants due to snowballing [13].

Triangulation

Three triangulation strategies were applied: a senior scientist (KM, PhD in sociology) listened to n=3 the audiotaped interviews to provide feedback to the interviewer (JFLK), n=2 interviews were double-coded (JFLK,KM), focused thematic summaries and results were discussed and discrepancies resolved iteratively among the junior researchers (BA, SS, GB, JFLK), who had different scientific backgrounds (healthcare science, medicine, economics and ethics). The senior scientists (KM, ECW, WG, JS) with different scientific backgrounds (sociology, medicine, ethics and economics) were part of the iterative process and provided guidance, feedback and evaluation.

Data availability

The focused thematic summaries are published [13] and available open access. The inductive categories are part of the results.

**Coding tree**

| **List of codes** |
| --- |
| reimbursement |
| no reimbursement |
| reimbursement, that does not completely cover costs |
| reimbursement, that exceeds the costs |
| covered costs for treatments with questionable cost-effectiveness |
| Financially incentivized actions |
| deprioritization |
| selection |
| cherry picking |
| lemon-dropping |
| rationing |
| implicit |
| explicit |
| prioritization |

References

13 König JFL, Aufenberg B, Brei G, et al. How do financial considerations influence medical decision-making? A qualitative-empirical study in German cancer medicine. *BMJ Public Health* 2025;3(2):e003346. doi:10.1136/bmjph-2025-003346 [published Online First: 27 November 2025]

16 Kuckartz U. Qualitative Inhaltsanalyse. Methoden, Praxis, Computerunterstützung, 4th edn. Weinheim, Basel: Beltz Juventa 2018.

27 Beauchamp TL, Childress JF. Principles of biomedical ethics. New York, Oxford: Oxford University Press 2013

37 Akyel D. Ökonomisierung und moralischer Wandel: Die Ausweitung von Marktbeziehungen als Prozess der moralischen Bewertung von Gütern.

Guidelines for expert interviews with physicians with controlling experience AP2 "Qualitative study of relevant decision-making situations"

**Introduction and personal introduction**

First of all, I would like to thank you for agreeing to support us and participate in this interview. You have already been informed about the content

and the aim of the study. I would like to repeat a few points and briefly explain the structure of our conversation.

But first, a few words about myself. My name is Julia König and I am a researcher and physician specializing in internal medicine at the National Center for Tumor Diseases at Heidelberg University Hospital. For this study, I am conducting interviews with approximately 15 physicians with experience in controlling.

**Information about the study**

As you have already been informed, in this study we are interested in how the treatment of cancer patients has changed as a result of financial and economic influences and what the effects of these changes are. The term "economization" is often used in this context, which can have both positive and negative connotations.

Our aim is to identify specific decision-making situations that particularly highlight the financial influences on the healthcare system. Based on these decision-making situations, we intend to create an empirical baseline of the financial influence on the treatment of cancer patients. Finally, the project aims to develop recommendations on how to deal with financial influences in the treatment of cancer patients.

If you have any questions during the interview, please do not hesitate to ask again or to clarify anything.

**Information about the interview**

For this study, we rely on your personal experiences and perceptions. It is important for us to learn and understand which issues are relevant to you when you think about possible financial influences on the treatment of cancer patients. So there are no wrong or right answers. We are interested in your subjective point of view.

During the interview, I will ask you various open-ended questions, and I would ask you to tell me everything that is important to you. Please take as much time as you need to answer the questions. I estimate that the interview will take about 40-60 minutes. You can end the conversation at any time.

The interview will be recorded with an audio device. This will assist us in the subsequent analysis. Do you agree to this?

**Information on data protection**

• I would like to reassure you that participation in the interview and answering the individual questions is voluntary.

• We will, of course, treat the interview material as strictly confidential and pseudonymize it. This means that all personal data about you that could be traced back to you will be deleted or pseudonymized, i.e., encrypted. The audio record will be deleted as soon as we have transcribed the interview.

Do you have any questions about the content of the study or the interview process?

Then I will now turn on the audio device. Turn on the recording device

**Introduction** (goal: build trust; explore possible topics)

| Invitation to talk |  | Further questions |
| --- | --- | --- |
| 1. What is the first thing that comes to mind when you think about economic influences in the treatment of cancer patients? | | • Can you give me an example from everyday life?  • In other words...?  • Take your time to think about it.  • And further?  • What else can you tell me about this?  • You said earlier... To come back to topic X... |
| 1. In your opinion, which decisions in cancer medicine are subject to economic influences? | |  |

**Topic block I) Influences and incentives** (Goal: Identifying influencing factors and incentive systems)

| Narrative request |  | Further questions |
| --- | --- | --- |
| 1. Can you think of any situations from your everyday clinical practice in which the treatment of patients differed from what it should have been for medical reasons due to economic factors? | | • Examples that comply with guidelines?  • Can you give me a specific example from everyday life? : Administration of drugs; outpatient vs. inpatient sector, bed capacity, staffing, time management |
| 1. Which economic influences do you consider appropriate and which inappropriate? | | • Specific cases or situations? |
| 1. Where do you think conflicts arise between what is medically needed and financial incentives?    1. Where is consultation time reimbursed in the outpatient/inpatient/day-care system? | |  |
| 1. Which reimbursement strategies do you perceive as strong financial incentives? | |  |
| 1. When do you ignore reimbursement structures or financial incentives? | |  |

**Topic block II) Requirements** (goal: identification of misguided practices and relevant cases)

| Narrative request |  | Further questions |
| --- | --- | --- |
| 1. Where do reimbursement structures lead to misguided patient management? | | • different sectors  • Lucrative procedures  • What else can you tell me about this? |
| 1. In which case constellations do you think this influence is particularly significant?    1. How is follow-up care reimbursed in outpatient/inpatient/day-care settings?    2. Where do you see problems with the reimbursement of oral and subcutaneous forms of administration?       1. Are there any drugs that are particularly affected?    3. How do you deal with the fact that the same therapeutic preparations are reimbursed differently depending on the form of administration? | | • lucrative vs. less lucrative procedures, outpatient vs. inpatient sector, "fit" vs. "less fit" patients |
| 1. What strategies are there for dealing with the conflict that arises from these requirements?    1. How do you deal with the reimbursement conditions for complex palliative care? | | • Can you give me an example from everyday life? : Case mix index, case numbers, efficiency goals, financing |

**Topic III) Revenue and losses** (goal: identifying sources of revenue and loss traps)

| Narrative request |  | Further questions |
| --- | --- | --- |
| 1. Which reimbursement structures lead to losses in cancer medicine? | | • Can you give me an example from everyday life?  • In other words...?  • Take your time to think about it.  • And further?  • What else can you tell me about this?  • You said earlier... To come back to topic X... |
| 1. In which cases is it clear from the outset on that financial loss will have to be accepted?    1. Are there any drugs that are too expensive for inpatient use? | |  |
| 1. How do you try to compensate for these losses? | |  |
| 1. Which reimbursement structures lead to sufficient income in cancer medicine, and what are the reasons for this?    1. How can revenue still be generated if there is no DRG for the case? | |  |
| 1. Are there "lucrative" patients in cancer medicine? | |  |

**Topic block IV) Positive effects** (goal: improving medical care through economic influences)

| Invitation to share |  | Further questions |
| --- | --- | --- |
| 1. In your opinion, where do economic conditions have a positive influence on the treatment of cancer patients? | | • Specific cases or situations? |
| 1. What examples have you seen where financial considerations have made the treatment of cancer patients more efficient? | |  |
| 1. Where do you think there are still buffers against unnecessary use of resource in the system? | |  |
| 1. What do you take from your experience in controlling and apply to daily practice, and what do you pass on to the team? | | • Changes in medical practice due to billing practices |

In the last section, I would like to ask you what changes you personally would like to see in the system.

**Topic block V) Potential for change** (goal: identifying desired outcomes and potential for change)

| Invitation to share |  | Further questions |
| --- | --- | --- |
| 1. There are currently political efforts to change the inpatient reimbursement system. What changes would you like to see there? | | • Subjective suggestions for improvement  • staff  • Prioritized changes  • Subjective suggestions for improvement |
| 1. In your opinion, which economic incentives should be abolished? | |  |

We are now coming to the end of this conversation. Finally, I would like to give you the opportunity to share something with us—some advice, a concern, or a wish.

**Concluding phase** (goal: allow space for unspoken thoughts, summarizing)

| Invitation to share |  | Points of content |
| --- | --- | --- |
| 1. Is there anything else you would like to share that we have not addressed so far? | | - Unspoken - Additions |

Thank you very much for this conversation, your openness, and your willingness to share. If you have any further questions about the course of study, please feel free to contact us.

**Stop recording**
